# Exploring the Relationship Between Gingivitis and Hypothyroidism in Children

**DOI:** 10.31661/gmj.v13i.3409

**Published:** 2024-06-20

**Authors:** Andisheh Amini, Pouria Farahani, Mahsa Etemadi, Pardis Khoshnood, Asieh Mozaffari, Bahareh Sanaee

**Affiliations:** ^1^ Department of Pediatrics, School of Dentistry, Isfahan University of Medical Science, Isfahan, Iran; ^2^ Shahid Beheshti University of Medical Science, Faculty of Dentistry, Tehran, Iran; ^3^ Department of Periodontology, School of Dentistry, Tehran University of Medical Sciences, Tehran, Iran; ^4^ Department of Restorative Dentistry, School of Dentistry, Ahvaz Jundishapur University of Medical Sciences, Ahvaz, Iran; ^5^ Department of Periodontics, Faculty of Dentistry, Qazvin University of Medical Sciences, Qazvin, Iran; ^6^ Department of Pediatric Dentistry, Faculty of Dentistry, Mashhad University of Medical Sciences, Mashhad, Iran

**Keywords:** Gingivitis, Hypothyroidism, Thyroid Hormones, Oral Health, Inflammation, Interdisciplinary Care

## Abstract

Periodontitis common oral health problem for children, which involves the inflammation of gum tissue. Hypothyroidism, which is a systemic disorder characterized by the decrease in thyroid hormone levels, has its patients suffering from many dysfunctions in the body. In spite of the fact that they apparently belong to distinct spheres, recent research looks suspiciously for a probable correlation between the two diseases, i.e. gingivitis and hypothyroidism. This paper is a comprehensive narrative review that focuses on explaining the unique relationship between these two conditions in children as it relates to the mechanisms, clinical manifestations, diagnostic difficulties, and therapeutic approaches. Through the process of literature review syntheses, previously unknown interrelations between hypothyroidism and gingival health are discovered. Therefore, the multidisciplinary approach may be one of the factors that improve the overall condition of the patients with these comorbidities as well as the effectiveness of the treatment due to the partnership through the cooperation between dental and endocrine specialists.

## Introduction

Gingivitis and hypothyroidism are two prevalent health issues affecting both children and adults that have been extensively studied over the past decade. Although gingivitis is primarily recognized as a threat to oral health, research has identified its effects beyond the mouth [1][2]. On the other hand, hypothyroidism is known to have widespread effects on the body’s physiology [3]. Historically, these two conditions have been seen as separate; however, emerging evidence suggests potential connections between them [4][5][6][7]. There is an established correlation of these conditions with the body's elevated levels of inflammation. In the case of gingivitis, the inflammation occurs only in the vicinity of the gums, but in hypothyroidism it can affect almost any organ or tissue [8][9][10]. With respect to clinical manifestations, hypothyroid pediatric patients may suffer from a number of oral health problems that can lead to such problems as gingivitis. This can be explained by the fact that the thyroid gland is responsible for maintaining the oral cavity in a healthy state [11][12][13][14]. When the gland of thyroid becomes underactive, as it is in cases of hypothyroidism, can cause a reduction of the saliva production that could be one of the factors of gingivitis [3][15]. Besides that, hypothyroidism can interact with the immune system adversely, rendering patients with gingivitis more prone. Distinguishing the gingivitis in the dysfunction patient's with hypothyroidism is a difficult task since the symptoms of both conditions share a similar picture [2][14][16].

One research showed that hormone treatment of hypothyroidism has an effect on gingivitis. The results of the study showed that thyroid hormone replacement therapy is not only effective in correcting the thyroid dysfunction but also on periodontal health [17]. In particular, the treatment led to the decrease of gingival inflammation and positive changes in clinical parameters of periodontium, pointing to the presence of systemic relationship between thyroid hormones levels and periodontal condition [17][18]. When comprehending the mechanisms that are behind, clinical presentations, diagnostic difficulties and therapeutic position of this association, healthcare professionals provide better healthcare to the patients with these conditions [11]. This review aims to explore the underlying mechanisms, clinical manifestations, diagnostic challenges, and therapeutic implications of gingivitis in Pediatric patients with hypothyroidism.

## Correlation Between Gingivitis and Hypothyroidism

The exact prevalence of gingivitis in hypothyroid patients is not clearly quantified in the available literature [11]. However, Venkatesh Babu, et al., [10] revealed that children with thyroid disorders dental caries and periodontal diseases are significantly higher as compared to healthy children [10]. So, more well-designed studies are needed to determine the strength of the association and the impact of thyroid hormone replacement therapy on periodontal health in these patients [11][19]. Gingivitis and hypothyroidism have become the recent focus of healthcare research as health scientists pay attention to how these diseases affect the lives of affected individuals and may also be associated in their epidemiology and the manner in which they interact [7][11].

Gingivitis conquers the position of the most widespread oral health disease worldwide, involving people both adults and children, as well as specific population groups. Research has proven again and again that gingivitis affects a high number of people; measurements go as far as suggesting a large number of the population suers gum inflammation [20]. Multiple factors play a role for the emergence and aggravation of gingivitis including the lack of proper dental hygiene, smoking, diabetes and short genetic features [20][21].

As expected, based on the nature of related fields, recent studies associate the gingivitis with hypothyroidism. An important shared risk factor is smoking and systemic inflammation, which effectively increases the possibilities to have these conditions at the same time [6][11][22]. Besides, such hormones also play the key role in managing immune responses and tissue repair which, therefore, may affect the inflammatory processes of gingivitis in a critical way.[23][24]

The epidemiological link between gingivitis and hypothyroidism as complex as well as multi-layered has been comprehended by the different studies providing the vision of the possible associations between the two conditions [11][25][26]. On this basis, it can be considered that the level of hypothyroidism may be higher among people with desquamative gingivitis but the researchers did not account for other probable confounding factors [27]. The two major diseases in children are gingivitis and hypothyroidism and should not be taken lightly because of the long-term effects that the diseases have on the human body. Periodontitis develops from gingivitis that is an inflammation of the gum that results from the growth of the sticky film called the plaque. Such inquiry and gingivitis can result to chronic periodontal diseases hence a child’s dental health and quality welfare is highly compromised [6][11].

According to Léger et al. [18] early diagnosing of this condition is vital since it helps in early management to avoid growth and cognitive impairment in affected children. A relationship exists between gingivitis and hypothyroidism since hypothyroidism directly affects the sensitivity, immunity and metabolism of the body to increase inflammation of the gingiva. This means that hypothyroid states will directly affect the immune response and will render children vulnerable to infections and chronic inflammation [11][28].

Moreover, The decline in serum thyroid-stimulating hormone (TSH) levels within the normative spectrum among hypothyroid patients subsequent to hormone supplementation is attributed to the diminishment of inflammatory markers implicated in both periodontal and thyroid dysfunction [17]. Therefore, it is crucial to explore how these conditions are connected and how pediatric care providers can help create a functional link between them so that the two fields of human health, dentistry and endocrinology, can work towards decreasing poor outcomes in the long run.

## Underlying Mechanisms Linking Gingivitis and Hypothyroidism

**Figure-1:**
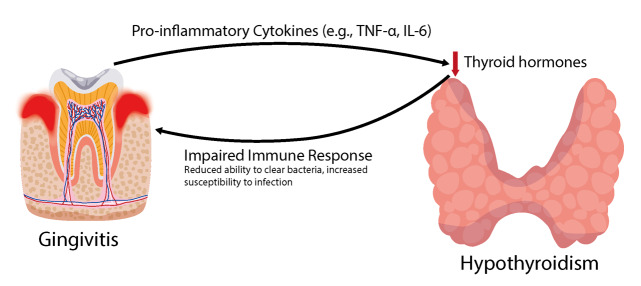
Figure 1. Schematic Illustration of Underlying Mechanisms Linking Gingivitis and Hypothyroidism

Immune System Modifier and Inflammation

Immunologic response and inflammation are closely linked, and they are predominant in the development of gingivitis and hypothyroidism. Gingivitis may be defined, primarily, as an inflammation affecting the gum tissue due to bacterial accumulation on the teeth. In response to this bacterial hostility, the host immune system produces a number of pro-inflammatory cytokines such as the tumor necrosis factor-alpha (TNF -α) and the interleukins (e. g. g. , IL-1β, IL-6). These cytokines are central to the inflammation process that if persistent causes tissue pathology and periodontal disease [8][29][30][31].

In some cases, immunological processes play key role in hypothyroidism, for example, autoimmune thyroiditis, or Hashimoto’s thyroiditis where immune system dysfunction is often observed in hypothyroidism patients. It involves production of antibodies against thyroid peroxidase and thyroglobulin leading to inflammation of thyroid gland and consequently low production of thyroid hormones. The pathological conditions that are known to develop in autoimmune thyroiditis; chronic inflammation may lead to the inflammation of gingiva through the following reasons [2][29][32]. Firstly, the immune system is being affected in hypothyroid; this is an implication that the body is going to offer poor resistance to oral pathogens hence poor control of bacterial colonization in the gingival tissues. Secondly, the typical chronic low-grade inflammation in hypothyroid patients can increase the levels of pro-inflammatory cytokines. These cytokines can further increase the level of inflammation in the gingival tissue promoting gingivitis and may also escalate the periodontal diseases. From this bi-directional relationship, it is therefore imperative that, systemic inflammation be given attention in overall control of oral health [29][33].

Role of Thyroid Hormones in Gingival Health

T4 thyroid hormone and T3 are essential hormones that relate to the metabolism processes ant are also related to cell division, tissue development and tissue regeneration. Such hormones are involved in activities that relate to the health of the gingiva in human beings [23][34][35]. In hypothyroidism, deficiency of T4 and T3 causes moderate reduction in necessary metabolic changes which can potentially be detrimental to the gingiva. The decrease of T3 and T4 elevates blood levels show lower rates of the metabolic and regenerative processes in the mucous membranes of the gingiva. This impairment can be manifested as; prolonged or delayed wound healing, decreased synthesis of collagen fibrils, and tissues of the gingival epithelium. This weakens the gingiva making it predisposed to infection and accumulation of bacteria thus the creation and persistence of gingivitis [9][35][36].

Moreover, the thyroid hormones play vital role in the regulation of blood circulation including the blood supply to tissues including gingival tissues. Appropriate blood flow is required in order to allow nutrient and immune cells to reach the goal tissues required in sustaining the gingival tissues’ health and prohibiting bacterial intrusion [23][35][36][37] (Figure-1).

Influence of Systemic Inflammation on Thyroid Hormones

Chronic gingivitis is closely connected with hypothyroidism as inflammation can greatly affect this gland’s functionality. Since periodontal disease is a state of chronic inflammation, the participants diagnosed with the mentioned condition had increased TNF-α and IL-6 level in the systemic bloodstream. These cytokines are thus capable to have an ability to so regulate the biosynthesis of the thyroid hormones or otherwise the overall status of the thyroid hormones in the system. They are a group of proteins that either incite inflammation or control the hypothalamic-pituitary-thyroid (HPT) axis which is involved in the release of thyroid hormones. IL-6 also may interfere with the secretion of hypothalamic TRH and pituitary TSH.

This disruption is most probable to trigger a reduction in the stimulation of the gland to secrete T4 and T3 hormones in thyroid gland. In addition, inflammation also tends to alter the oxidant state, which in turn affects the thyroid gland adversely [36][38].

Moreover, it has also been linked with states related to inflammation and increased levels of oxidative stress load. The tissue of the thyroid may also produce ROS because inflammation may persist leading to alteration of the tissue components and the DNA to cause hypothyroidism. Apart from this, the already fragile effects of systemic inflammation on thyroid further leads to the vicious cycle of gum and thyroid diseases [8][38].

Specifically, it is understood that inflammation is the common link between gingivitis and hypothyroidism, as well as changes in the immune system and sex hormones. In order to understand these pathways, it is necessary to define the size that appears in diagnosis and treatment options in case of working with patients who have gingival and thyroid diseases [9][34][35].More research is needed in this area to have better knowledge of these mechanisms and to develop an efficient strategy for tackling these intricate and interrelated diseases.

## Diagnostic and Therapeutic Challenges

**Table:** **Table 1.** Diagnostic and Therapeutic Challenges in Coexisting Gingivitis and Hypothyroidism

**Challenges**	**Description**
**Overlapping Symptoms**	Patients may present with symptoms common to both conditions, such as fatigue and dry mouth, making it challenging to differentiate between gingivitis and hypothyroidism.
**Subclinical Presentations**	Subclinical hypothyroidism can manifest with mild or nonspecific symptoms, necessitating comprehensive evaluations to detect underlying thyroid dysfunction.
**Coordinated Care**	Effective management requires collaboration between dental and medical professionals to ensure comprehensive evaluations and cohesive treatment plans.
**Impact on Oral Treatments**	Hypothyroidism can affect the response to periodontal treatments, requiring adjustments to treatment protocols to optimize outcomes.
**Medication Interactions**	Potential interactions between thyroid medications and drugs used in dental procedures must be considered to prevent adverse effects and ensure treatment efficacy.
**Lifestyle and Behavioral Factors**	Addressing lifestyle factors such as poor oral hygiene and smoking cessation is essential for managing both conditions effectively and preventing disease progression.
**Long-term Management**	Continuous monitoring and regular follow-up appointments are necessary to manage the chronic nature of both gingivitis and hypothyroidism effectively.
**Children Compliance**	Educating Children about the importance of adherence to treatment plans and lifestyle modifications is crucial to achieving optimal health outcomes.

Gingivitis and hypothyroidism can be very challenging to diagnose and treat because of the connection of oral health to systemic health. Many cases can be misleading since several diseases may exhibit the same signs and symptoms like fatigue and dry mouth [11][26][31]. More so, since hypothyroidism may also present itself in subclinical form, it is advisable that an overall dental and medical check-up be conducted [15].

Diagnosis of the condition involves combination of clinical assessment and laboratory investigations such as thyroid profile and periodontal assessment probing. It is therefore important for dentists and physicians to work in tandem in order to manage such diagnostic complexities [11][15][25].

Table-1 showed common diagnostic and therapeutic challenges in coexisting gingivitis and hypothyroidism. Managing patients affected with gingivitis and hypothyroidism needs comprehensive care to cure both the localized and generalized conditions [11]. Therapeutic complications stem from external circumstances that affect oral administrations, interactions of the drugs, and overlooking lifestyle and behavioral patterns that exacerbate diseases. Special attention should be paid to medication and dosing, which is explained by the ability of thyroid hormone therapy to affect periodontal therapy and interact with drugs used in dentistry [15][22].

## Future Directions and Research Recommendations

Further studies on the correlation between gingivitis and hypothyroidism in children should focus on the following key objectives to provide better outlooks that will support the clinical reality and therapeutic action. Prospective investigation is paramount to understand outcomes of hypothyroidism on the periodontal conditions from early childhood to adolescence.

Such studies would contribute to gaining valuable knowledge about the dynamics of periodontal diseases in relation to thyroid hormone deficiencies as well as to determining time points for prevention [25][39].

Community education campaigns should focus on routine thyroid tests and early intervention to address hypothyroidism in kids. By creating interventions for parents and healthcare professionals, those at risk for developing poor oral health habits may be more inclined to seek preventive measures managed systematically. There is, therefore, a need for cooperation between the pediatric endocrinologists and dentists in order to integrate their care practices in order to tackle endocrine and periodontal problems in children [18].Consequently, understanding the connections between gingivitis and hypothyroidism in children focus on clinical research, molecular biology, and demographic health solutions. By employing such strategies, it will help in the understanding of these conditions and improve patient outcomes by focusing on patient centered care.

## Conclusion

Therefore, the reciprocal relationship between gingivitis and hypothyroidism suggests that more investigation into the etiopathology mechanisms and an evidence-based approach to treating these conditions is needed. In the course of our discourse, we have admitted a more nuanced bi-directional cross talk between gingivitis and thyroid gland activity using cytokines, hormonal agents, and inflammation. Despite this, many things are unanswered regarding these distinctions and ought to be explored in order to refine diagnostics and to optimize therapeutic practices [23][31][40].

Thus, the future endeavors should involve further interdisciplinary approaches and translational investigations to address the diagnostic and therapeutic demands arising from composite gingivitis and hypothyroidism states. Therefore, in theoretical and practical aspects, there should be a dental and medical approach to define a more complete vision of how oral and overall health are connected. The further research should continue to explore the nature of the link between these disorders, to create targets for motives of intervention and carry out prospective studies to consider the causal relationships and efficacy of the treatment strategies [26][31].

Thus, lastly, it may be crucial to increase awareness of the link between gingivitis and hypothyroidism in order to improve the patient care and health outcomes, as well as to reduce the disease burden for patients suffering from both conditions [11].

Further advancement in research and clinical works should be undertaken to accommodate changing demands of the patient with gingivitis and hypothyroidism; patient centered approaches in oral and systemic health care should be introduced.

The link between gingivitis and hypothyroidism in children is complex and requires increased research and clinical interest. Gingivitis, which is usually the first stage of periodontal disease, poses a great threat to oral and general health if not controlled [9][39].

the chronic oral diseases and inflammation such as gingivitis might have negative effects on overall health and worsen endocrine disorders. This reciprocal interaction requires an integrated assessment and treatment of the two diseases. Some studies should be encouraged in order to compare the long-term impacts of hypothyroidism on periodontal health and periodontal disease on hypothyroidism [10][41].

Moreover, Bhankhar et al., [17] reported The treatment of hypothyroidism can ameliorate periodontal conditions by decreasing inflammatory markers, which in turn affects thyroid hormone levels. Consequently, the immune system acts as a crucial intermediary connecting thyroid dysfunction and periodontal diseases. Léger et al. [18] demonstrated that it is crucial to include dental check-ups in children’s hypothyroidism care plans and to assess thyroid function in pediatric patients with chronic gingivitis. Such integrated healthcare strategies could enable early diagnosis, prevention, and control of such related ailments which would in the long run enhance patients’ health and well-being.

Ultimately, emphasizing the interrelation between gingivitis and hypothyroidism in pediatric patients and fostering interdisciplinary, evidence-based research along with collaborative care strategies is essential. These initiatives will enhance the comprehension and management of these conditions, thereby promoting improved health outcomes and developmental progress in affected children.

## Conflict of Interest

None declared.
